# The Breakdown of Neurovascular Barriers: Molecular Mechanisms of Tight Junction Dysfunction

**DOI:** 10.1007/s12035-025-05592-z

**Published:** 2025-12-09

**Authors:** Sowmya Shree Gopal, Mandeep Kaur, Sophie Lanzkron, Amit K. Srivastava

**Affiliations:** Department of Medicine, Cardeza Foundation for Hematologic Research, Sidney Kimmel Medical College, Thomas Jefferson University, Philadelphia, PA USA

**Keywords:** Tight junctions, Blood-brain barrier, Blood-spinal cord barrier, Barrier permeability, Neurovascular dysfunction

## Abstract

The central nervous system (CNS) relies on tightly regulated barriers to maintain homeostasis and protect neural tissue from blood-borne toxins, pathogens, and inflammatory mediators. Tight junctions (TJs) are critical components of the blood-brain barrier (BBB) and blood-spinal cord barrier (BSCB), forming selective paracellular seals that regulate molecular trafficking. These structures comprise transmembrane proteins and cytoplasmic scaffolding proteins, which anchor TJs to the actin cytoskeleton. The spatial organization and function of TJs are dynamically regulated by calcium-dependent signaling, phosphorylation events, and G-protein-mediated pathways, which govern their assembly, disassembly, and response to physiological and pathological stimuli. The integrity of TJ complexes is particularly vulnerable to disruption in neurological disorders. Dysregulation of key TJ proteins has been implicated in neurodegenerative diseases, neuroinflammation, and CNS injury, leading to barrier permeability defects that exacerbate disease progression. Emerging therapeutic strategies aim to modulate TJs to stabilize barrier integrity and to mitigate pathology. This review examines the molecular architecture and regulatory mechanisms of TJ complexes, their dysfunction in disease states, and the translational potential of targeting them for therapy. A detailed understanding of TJ dynamics is essential for developing strategies to restore barrier function in neurological disorders.

## Introduction

Neurological disorders resulting from intrinsic dysfunctions within the central nervous system (CNS) or its interactions with the surrounding microenvironment represent a growing and multifaceted global health challenge. These conditions affect individuals across all stages of life and are prevalent in both developed and developing regions. According to the 2021 Global Burden of Disease, Injuries, and Risk Factors Study (GBD), supported by the World Health Organization (WHO), over 3 billion people worldwide are affected by neurological conditions, making them the leading cause of illness and disability globally. Approximately 1.5 billion individuals currently live with CNS disorders, which contribute to 1% of global deaths and 11% of the overall disease burden. Given the aging global population, these figures are projected to rise, highlighting the urgent need for effective and innovative therapies to address these conditions [1–4].

**Fig. 1:**
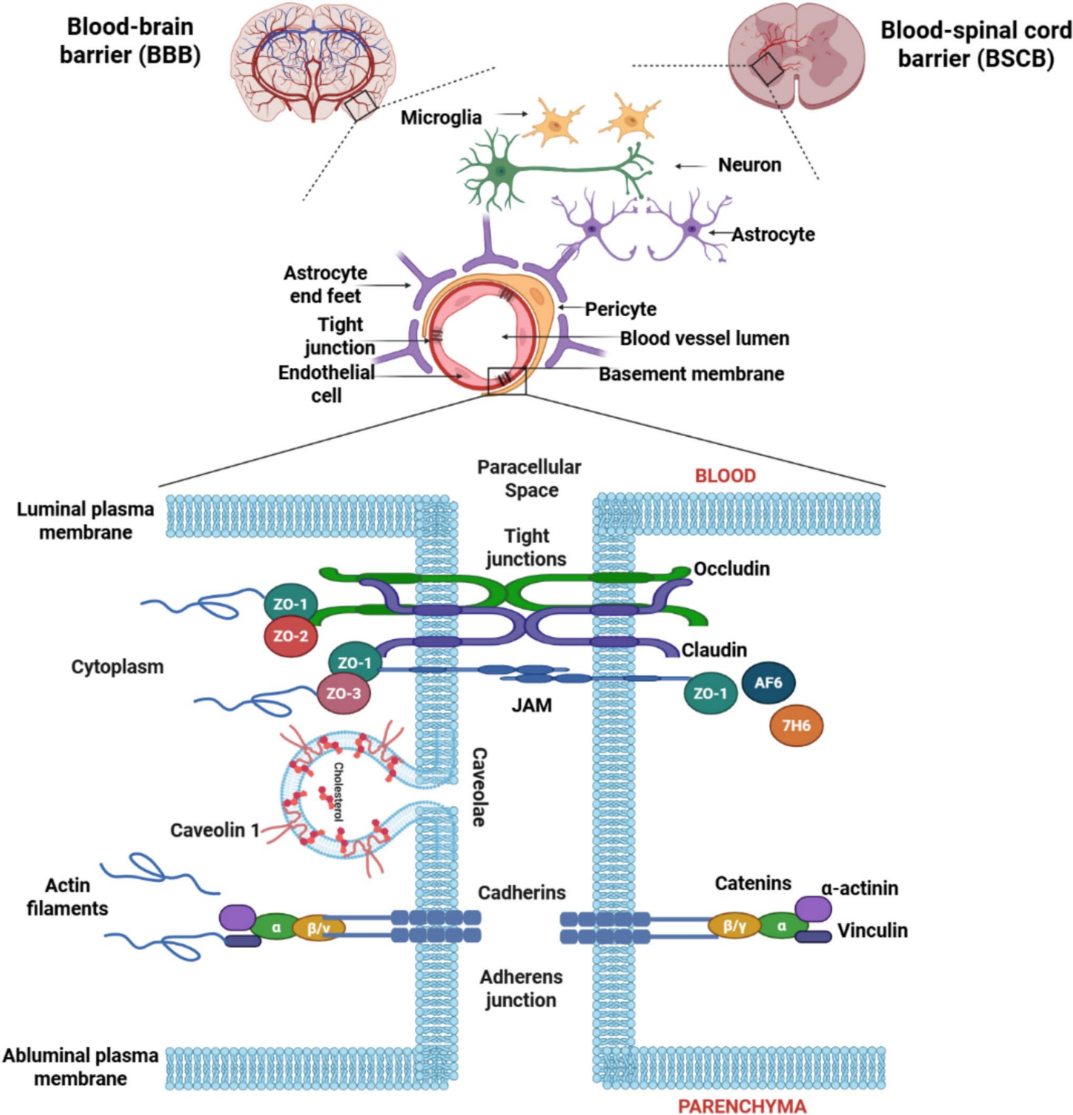
Junctional complexes of the BBB and BSCB

Central to the pathophysiology of many neurological disorders are disruptions in the blood-brain barrier (BBB) and blood-spinal cord barrier (BSCB), both of which serve as crucial protective interfaces regulating the exchange between the bloodstream and the central nervous system (CNS). These barriers are primarily formed by endothelial cells connected through tight junctions (TJs) and adhesion proteins **(**Fig. 1**)**, which work together to prevent harmful substances such as pathogens and toxins from entering the CNS [5]. Similarly, the blood-retinal barrier (BRB) functions as a highly selective protective system analogous to the BBB. It is composed of specialized TJs within retinal capillary endothelial cells and retinal pigment epithelial cells (RPE) that maintain the immune privilege and homeostasis of the retina [6]. The BBB and BSCB are essential in maintaining CNS homeostasis by controlling the selective passage of molecules, ions, and cells, thus safeguarding neuronal function and CNS integrity. The neurovascular unit (NVU), which consists of endothelial cells, astrocytes, pericytes, neurons, microglia, and the acellular basement membrane, regulates barrier function and supports vital processes such as neurovascular coupling, angiogenesis, and neurogenesis [7–9].

TJs located at the apical membranes of endothelial cells are crucial for maintaining the integrity of the BBB and BSCB. These junctions restrict the paracellular diffusion of solutes and ions, preventing the mixing of luminal and abluminal compartments. Dysfunction in these TJs plays a significant role in the pathogenesis of various neurological disorders. Despite their similar roles in the BBB and BSCB, recent studies have revealed regional differences in blood-CNS permeability, suggesting that the two barriers exhibit distinct properties depending on their location along the neuroaxis [10–13].

The integrity of both the BBB and BSCB is critical for developing effective therapies for neurological diseases. Disruption of these barriers can not only lead to severe pathology but also hinder the delivery of therapeutic agents to the CNS. Existing literature on the role of CNS TJ proteins has predominantly focused on their involvement in BBB and BSCB permeability alongside their cellular mechanisms. However, understanding their contribution and their interrelationships under normal and pathological conditions of neurological disorders is scarce. This review article focuses on the role of TJs in maintaining the integrity of CNS barriers, examining how these junctions are regulated during both homeostasis and disease. Moreover, we discuss emerging therapeutic strategies that aim to modulate tight junctions as potential treatments for neurological disorders, highlighting their potential to be targeted in clinical interventions.

## Role of CNS TJs in the Regulation of BBB and BSCB Homeostasis

The discovery of TJs began with their initial visualization by electron microscopy in 1963, when Farquhar and Palade identified the zonula occludens as a distinct cell-cell adhesion structure between epithelial cells [14]. Early functional insights linked these structures to paracellular transport regulation. In 1971, Hashimoto et al. further provided ultrastructural evidence of TJs in human epidermis through lanthanum tracer studies [15]. Molecular characterization advanced significantly in the 1980 s and 1990 s, highlighted by the identification of zonula occludens-1 (ZO-1) in 1986 as the first scaffolding TJ protein, and occludin in 1993 as the first transmembrane TJ protein associated with barrier function [16]. The discovery of claudins in 1998 by Furuse and Tsukita identified a family of integral membrane proteins forming the backbone of TJ strands and critically defining TJ permeability properties [17]. Together, these foundational studies established the ultrastructural, functional, and molecular framework that defines TJs today as dynamic protein complexes essential for barrier integrity.

TJs undergo structural and functional modifications to balance the need for maintaining a selective seal while permitting controlled paracellular transport under varying physiological and pathological conditions. They play a key role in regulating the homeostasis of both the BBB and the BSCB. Alterations in TJ protein expression or post-translational modifications can compromise BBB integrity and function, reflecting changes in junctional protein behavior [18]. Therefore, numerous factors and mechanisms govern TJ assembly to ensure optimal barrier maintenance and functional stability [19, 20]. Core TJ components include claudins, occludin, and junctional adhesion molecules (JAMs). These transmembrane proteins interact with cytoplasmic scaffolding proteins such as ZO-1, ZO-2, and ZO-3, which are essential for TJ assembly, localization, and signal transduction. ZO proteins belong to the membrane-associated guanylate kinase (MAGUK) family and directly bind transmembrane TJ proteins like occludin and claudins, anchoring them to the actin cytoskeleton to regulate TJ architecture and function [21–25]. Additional accessory proteins in the BBB include cingulin, AF-6 (afadin), and the 7H6 antigen [18]. AF-6 (205 kDa) has two Ras-binding domains, one ZO domain, and multiple homologous areas with myosin V and kinesin. AF-6 is a scaffolding protein that regulates cell-cell contacts by interacting with ZO-1 at the N terminus Ras-binding domain [26]. Cingulin (140 kDa) is another scaffolding protein, linking TJ accessory proteins to the cytoskeleton. It interacts with other TJ proteins, such as ZO‐2, AF‐6, JAM, skeleton protein F‐actin, and other cingulin molecules [27]. Many TJs (ZOs, occludin, claudins) contain binding sites for actin, the primary cytoskeletal protein [28]. The 7H6 antigen protein (155 kDa) contains a putative ATPase domain for reversibly dissociating from the TJ under conditions of ATP depletion. This cytoplasmic accessory protein plays a significant role in regulating paracellular barrier function [29].

Additionally, tricellular junctional molecules such as tricellulin and the lipolysis-stimulated lipoprotein receptor (LSR or angulin 1) coordinately maintain the epithelial and the endothelial barrier integrity [30]. Under inflammatory conditions, the localization and the expression of both tricellulin and LSR are hampered, inducing compromised barrier function and upregulated leukocyte transmigration [31, 32]. Studies suggest that pro-inflammatory cytokines such as tumor necrosis factor (TNF-α) and interleukin-1β (IL-1β) alter the expression and localization, thus compromising the barrier function [32]. Furthermore, junctional adhesion molecule-like (JAM-L), a member of the JAM family, is actively regulated during inflammation. Through its interaction with α4β1 integrins on immune cells, JAM-L promotes trans-endothelial migration and immune cell adhesion [33, 34].

Beyond tricellular junctional molecules, the TJ complex also comprises various scaffolding and transmembrane proteins such as MAL and related proteins for vesicle trafficking and membrane link domain 3 (MARVELD3), the other member of the MARVEL domain-containing family [22], endothelial cell-selective adhesion molecule (ESAM), coxsackievirus and adenovirus receptor (CAR), and the MAGUK family, including ZO-1, collectively integrate barrier structural organization and signaling cascades of TJs [35]. MARVELD3 is involved in maintaining TJ assembly and cellular stress response [36, 37]. Parallelly, ESAM and CAR trigger intercellular adhesion and modulate endothelial permeability during inflammation [38]. Also, scaffolding proteins of the MAGUK family, including ZO-1, regulate the actin cytoskeleton and transmembrane TJ proteins. During inflammatory conditions, alterations in these molecules evoke junctional disassembly, cytoskeletal remodeling, and elevated paracellular permeability [38, 39].

Other signaling molecules, such as the caveolar protein caveolin 1 and extracellular calcium (Ca^2+^), also contribute to BBB integrity. Caveolae are the plasma membrane microdomains in endothelial cells that recruit TJ proteins to support barrier integrity [40]. Increased TJ permeability was seen in mouse brain endothelial cells due to the presence of inflammatory cytokine C-C motif chemokine ligand 2 (CCL2), which was found to be linked to claudin-5 and occludin internalization in a caveolae-dependent manner [41]. Ca^2+^ is required for the homotypic interaction of E-cadherin, which is thought to be the initial event of forming junctional complexes [42]. Conversely, intracellular Ca^2+^ plays a role in cell-cell contact, increased electrical resistance, ZO-1 migration from intracellular sites to the plasma membrane, and, notably, TJ assembly [43].

Cell adhesion molecules (CAMs) play a crucial role in maintaining endothelial cell integrity and regulate leukocyte trafficking under physiological conditions [44]. During homeostasis, CAMs such as intercellular adhesion molecule-1 (ICAM-1) and vascular cell adhesion molecule-1 (VCAM-1), and platelet/endothelial cell adhesion molecule-1 (PECAM-1), favor junctional stability and leukocyte surveillance [45]. Under inflammatory conditions, the proinflammatory cytokines such as TNF-α and IL-1β induced transcriptional upregulation of these CAMs through NF-κB and mitogen-activated protein kinase (MAPK) pathways [46]. Upregulated ICAM-1 and VCAM-1 expressions induce leukocyte adhesion and trans-endothelial migration, mediating immune cell recruitment [47]. Persistent activation of CAMs further disrupts TJs’ and AJs’ organization, leading to elevated endothelial permeability [48].

Phosphorylation of both transmembrane and accessory proteins plays an important role in establishing and regulating the TJ. Research has shown that extracellular stimuli rapidly cause TJ phosphorylation, promoting redistribution that results in TJ assembly or disassembly with modified barrier function, as most junction proteins have multiple phosphorylation sites [39]. Therefore, changes in the phosphorylation state of these proteins are an important regulator of permeability.

The protein kinase C (PKC) family of serine/threonine (Ser/Thr)-kinases plays a crucial role in regulating TJ permeability [49]. Occludin has Ser, Thr, and Tyr phosphorylation sites on its C terminus. It was observed that occludin phosphorylation on sites Thr-424/Thr-438 was required for phosphorylation on Tyr and Ser residues and attenuates occludin interaction with ZO-1, inducing barrier alterations and increasing paracellular permeability [50]. The phosphorylation of Ser, Thr, and Tyr residues also regulates ZO-1, as increased phosphorylation is associated with decreased expression of ZO-1. Ser/Thr kinase activity also regulates ZO-2, potentially increasing permeability. Various phosphorylation sites are present on claudin-5, with six observed kinase-specific phosphorylation sites and one site, Thr-207, that was found to affect TJ integrity [18]. Ser/Thr phosphorylation due to the activation of some Ser/Thr kinases is also associated with altered claudin-5 localization and expression, which further results in TJ complex disassembly [51]. While JAM-A is a transmembrane component of tight junctions, the specific effects of its phosphorylation on the endothelial barrier function are being investigated, with few studies suggesting a role in inflammation-induced barrier disruption [52]. Actin cytoskeletal components also have a role in barrier integrity. The RhoA/ROCK pathway regulates actin cytoskeleton rearrangements, but the myosin light chain kinase (MLCK) also plays a role, often in a coordinated manner. MLCK directly phosphorylates the myosin light chain, causing actomyosin contraction and endothelial barrier disruption. TJ integrity depends on the structural organization of actin [53]. ZO-1 can directly bind to claudin-1, occludin, other TJ proteins, and the actin cytoskeleton; however, these proteins do not always form a stable protein complex [54]. This suggests that a variety of factors can influence TJ protein dynamics. Moreover, one stimulus may not always affect the whole TJ assembly. The stimuli affecting occludin do not necessarily affect ZO-1 or claudin. Therefore, studying biochemical-functional relationships is essential to understand protein dynamics and paracellular permeability.

Other than phosphorylation, alterations in the expression and localization of TJ proteins can directly contribute to maintaining the barrier integrity. For example, TJs can relocate during diminished interactions. Stamatovic et al. showed that TJ relocation may not only directly affect barrier properties, but also modulate ongoing signaling processes [18]. Relocation can also result in new functions. If JAM-A relocates via macropinocytosis or translocation to the luminal membrane of endothelial cells, it can serve as a leukocyte adhesion molecule governing leukocyte infiltration in BBB dysfunction [55]. In pathological conditions, the disturbed organization of TJ assembly leads to increased barrier permeability or dysfunction [56, 57].

## Pathological Alteration of TJs in Various CNS Disorders

**Fig. 2:**
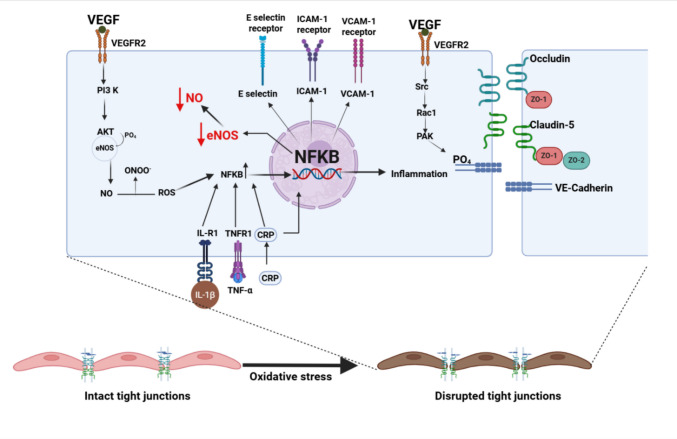
Oxidative stress and inflammation-mediated endothelial dysfunction and disruption of TJs

As mentioned earlier, TJ protein complexes are key regulators of CNS barrier properties. These complexes, however, are not static; they are highly dynamic structures whose expression and localization can be modulated in response to various pathophysiological stressors (Fig. 2).

In fact, the response to these stressors often leads to significant alterations in TJ complexes, oxidative stress, and inflammation, including the de novo synthesis of new proteins and the activation of distinct trafficking mechanisms [58]. These modifications can lead to the disruption of the protective barrier, promoting the onset and progression of various CNS disorders.

### Alzheimer’s Disease

The breakdown of the BBB is widely recognized as a crucial early indicator of Alzheimer’s disease (AD) [59]. A prominent feature of AD is the accumulation of amyloid-beta (Aβ) protein deposits, both in the brain parenchyma and within the walls of blood vessels. Additionally, the disease is characterized by neurofibrillary tangles, consisting of intraneuronal deposits of hyperphosphorylated tau, a microtubule-associated protein [60]. Several studies have highlighted the increased extravasation of plasma proteins in the brains of AD patients, further supporting the dysfunction of the BBB as an early indicator of AD [61–64]. Postmortem analysis of AD brains has shown a significant reduction in TJ proteins, such as claudin-5, occludin, and ZO-1, in cerebral blood vessels exhibiting amyloid angiopathy [65, 66]. However, a contradictory study [66] suggests that Alzheimer-type pathology in the cortex is not linked to the downregulation of claudin-5, occludin, or ZO-1.

Conflicting findings regarding BBB permeability and TJ modifications have also been observed in AD animal models. In Tg2576 mice, which overexpress the amyloid precursor protein (APP) with the Swedish mutations (K670N and M671L), researchers observed increased brain uptake of albumin and fluorescein, as well as increased extravasation of immunoglobulin G (IgG) and fibrin, indicating compromised BBB integrity [67–69]. Additionally, several studies have detected reduced levels of TJ proteins, including claudin-1, claudin-5, ZO-1, and occludin, in the brain microvessels of Tg2576 mice [66, 68–70]. Immunofluorescence labeling of ZO-1 and occludin in the cerebrovasculature of these mice revealed discontinuous or punctate staining patterns, suggesting weakened TJ integrity. Ultrastructural analysis of 5XFAD mice, which express mutant human APP with the Swedish, Florida (I716V), and London (V717I) mutations, as well as familial AD mutations in presenilin-1 (M146L and L286V), revealed significantly shorter TJs in the brain vasculature [71].

In contrast, no alterations were observed in BBB permeability to radiolabeled tracers ranging from 86 to 150 kDa in TauPS2APP mice, which express the German presenilin-2 mutation (N141I), the Swedish APP mutation, and overexpress tau [72]. These studies showed no changes in claudin-5 levels in both AD brain lysates and TauPS2APP mice. Similarly, research on 3xTG-AD mice, which express the APP Swedish mutant, mutated tau (P301L), and mutated presenilin-1 (M146V), found no changes in BBB permeability, although TJ proteins or integrity were not explicitly examined [73, 74].

Recent research has also explored the potential for modulating the gut microbiome to support neurovascular health. A human-origin probiotic cocktail was found to suppress gut permeability, thereby reducing inflammation and preserving BBB function. This, in turn, prevents pro-inflammatory burden on the brain, controls microglial activation, and mitigates Aβ accumulation [75]. This research highlights the potential of gut microbiome modulation as a therapeutic approach for preserving BBB integrity and preventing neurovascular dysfunction in AD.

### Epilepsy

Epilepsy, characterized by abnormal brain activity resulting in seizures, has been associated with several BBB abnormalities [76]. In both rat models of limbic epilepsy and human brain tissue obtained from individuals diagnosed with temporal lobe epilepsy, postmortem analyses have revealed extravasation of IgG and a significant loss of ZO-1 immunostaining, indicative of BBB compromise [77]. These alterations in BBB integrity were observed early following status epilepticus (SE) and during the disorder’s latent and chronic phases. Brain tissue from patients who succumbed during SE also exhibited albumin immunoreactivity around blood vessels, further supporting the link between BBB dysfunction and epileptic pathology [78]. In a rodent model of SE, increased permeability of brain capillaries to Texas Red was observed, accompanied by a marked reduction in TJ proteins, such as occludin, ZO-1, claudin-1, and claudin-5, within the BBB. These findings suggest that seizures contribute to TJ disruption. The release of glutamate during seizures activates cytosolic phospholipase A2, which in turn activates matrix metalloproteinases (MMP)−2 and MMP-9, both of which degrade TJ proteins and compromise BBB integrity [79].

Although poorer outcomes from epilepsy are linked to BBB dysfunction, the underlying molecular processes of BBB failure remain unknown. TJ proteins significantly regulate the integrity of the BBB. Claudin-5 is the most abundant in brain endothelial cells and controls size-selectivity at the barrier. Furthermore, it is still unclear how claudin-5 expression alteration relates to epilepsy despite being linked to illnesses such as depression, schizophrenia, and traumatic brain damage [80]. Studies of surgically resected brain tissue from patients with treatment-resistant epilepsy have demonstrated reduced expression of claudin-5, with corresponding dynamic contrast-enhanced magnetic resonance imaging MCAO revealing significant BBB breakdown [81]. Moreover, genetic heterozygosity for claudin-5 or targeted alterations in claudin-5 expression, specifically within the hippocampus, exacerbate kainic acid-induced seizures and induce BBB disruption. This research indicates that kainic acid triggers the downregulation of claudin-5, leading to the breakdown of BBB integrity. Furthermore, the inducible knockdown of claudin-5 in murine models results in severe neuroinflammation, increased mortality, and spontaneous recurrent seizures, highlighting the critical role of claudin-5 in maintaining BBB function and its potential as a therapeutic target in epilepsy [81].

### Hypoxia/Ischemia

In the microvasculature of the brain, intact TJs play a crucial dual role in regulating permeability and maintaining barrier integrity [82]. A thrombus or embolism-related restriction in the blood flow to the CNS is known as cerebral ischemia, and one of its main components is hypoxia. Increased BBB permeability has been linked to TJ changes in both hypoxia and ischemia [83–86]. Studies have suggested that BBB permeability following hypoxic or ischemic insult exhibits a biphasic pattern: an initial, continuous monophasic phase that occurs within hours of onset, followed by a prolonged phase that can persist for several days post-insult [84, 87, 88]. The extent and duration of these permeability alterations are influenced by multiple factors, including the type, severity, and duration of the occlusion, as well as species-specific differences and variations in detection methodologies. Notably, in female Sprague-Dawley rats, a hypoxic challenge (6% O₂ for 1 h) followed by 20 min of reoxygenation resulted in increased BBB permeability to (^14^C)-sucrose (~340 Da), while the permeability to Evans blue dye pre-bound to albumin (~80 kDa) remained unchanged, suggesting selective permeability changes favoring small molecules over macromolecules during acute hypoxia [84, 89, 90].

The increased permeability of the BBB under hypoxic conditions has been associated with disruptions in TJ protein structure and localization. Occludin, claudin-5, and ZO-1 exhibited altered distribution patterns following hypoxia, with occludin undergoing disruption of its high-molecular-weight oligomers [90–92]. Administration of the antioxidant tempol or the PKC inhibitor chelerythrine chloride was found to prevent these alterations, implicating reactive oxygen species generation and PKC activation in the regulation of BBB paracellular permeability during hypoxia-reoxygenation stress [90, 92]. Additionally, in murine models exposed to 8% O₂ for 48 h, increased BBB permeability to sodium fluorescein was associated with a significant reduction in occludin expression and alterations in occludin and ZO-1 localization [93]. These effects were mitigated by inhibition of MMP-9 or vascular endothelial growth factor (VEGF), indicating that MMP-9 and VEGF contribute to TJ disruption in ischemic conditions.

The presence of electron-dense TJs that effectively seal the inter-endothelial cleft in both directions contributes to the prevention of paraendothelial flow, thereby reducing infarct size and cerebral edema. Conversely, a less complex or dense TJ structure has been associated with an attenuated infarct process, suggesting that TJ integrity significantly influences ischemic outcomes [94]. The integrity of the BBB, the structural organization of TJs, and the expression and localization of TJ-associated proteins are influenced by claudin-3 [95]. Ischemic disturbances lead to periendothelial swelling of astrocytic end-feet, whereas a deficiency in claudin-3 results in endothelial injury but paradoxically reduces infarct size and edema formation [94].

The breakdown of the BBB following ischemia has been extensively documented in both experimental and clinical studies. In a rat model of transient middle cerebral artery occlusion (MCAO), an increase in BBB permeability to Evans blue was observed within 3 to 72 h of reperfusion, coinciding with significant reductions in occludin, claudin-5, and ZO-1 expression as well as visible disruptions in TJ complexes [96]. In a permanent MCAO model, a biphasic increase in BBB permeability persisted up to 120 h, correlating with marked reductions in occludin and claudin-5 [97]. Recent investigations have demonstrated that in ischemic stroke models, mRNA expression levels of claudin-1, claudin-3, claudin-12, and occludin were significantly reduced after 3 h of reperfusion, while claudin-5 mRNA levels were upregulated in the ipsilateral hemisphere relative to the contralateral hemisphere [94]. Notably, TJ length was reduced in the same study, and claudin-3 knockout mice exhibited decreased infarct size and reduced edema formation.

Several molecular mechanisms have been implicated in ischemia-induced TJ remodeling and BBB dysfunction. MMP activity is critical in regulating BBB integrity during ischemia, as MMPs degrade both extracellular matrix components and TJ proteins[98]. In a transient MCAO model, MMP-9 mutant mice exhibited significantly reduced Evans blue extravasation and ZO-1 downregulation but no changes in occludin levels [99]. Similarly, administration of a broad-spectrum MMP inhibitor (BB-1101) in hypertensive rats subjected to transient MCAO prevented increased BBB permeability to (^14^C)-sucrose and blocked the degradation of claudin-5 and ZO-1 [100]. MMP activation has been linked to nitric oxide signaling, as studies have demonstrated that nitric oxide-mediated downregulation of caveolin-1 triggers MMP activity [101]. Furthermore, inhibition of nitric oxide synthase using *N*^G^-nitro-l-arginine methyl ester (L-NAME) reduced ZO-1 downregulation and Evans blue leakage in transient MCAO rats [102]. Caveolin-1 knockout mice exhibited decreased expression of ZO-1, occludin, and claudin-5 while showing increased MMP activity and enhanced BBB permeability following photothrombotic ischemia [103]. Therapeutic interventions targeting BBB integrity have shown promise in experimental ischemic stroke models. Administering progesterone or allopregnanolone 1 h after occlusion was found to reduce Evans blue extravasation, inhibit MMP-2 and MMP-9 activation, and prevent the downregulation of claudin-5 and occludin [104]. Additionally, systemic inflammation was found to exacerbate BBB breakdown following ischemia, as transient MCAO in mice subjected to IL-1β administration resulted in sustained MMP-9 activation and claudin-5 rearrangement without affecting occludin levels [105]. Treatment with angiopoietin-1 following MCAO in rats attenuated Evans blue extravasation and modulated the downregulation of occludin and ZO-1 for up to 1 week post-ischemia [106].

Beyond TJs, adherens junction proteins (AJs), particularly VE-cadherin, are essential for maintaining endothelial barrier integrity. Hypoxic exposure significantly reduces VE-cadherin expression, leading to increased endothelial permeability. In cultured brain endothelial cells, hypoxia resulted in a reduction in transendothelial electrical resistance and an increase in sodium fluorescein migration, indicating barrier dysfunction. The study also revealed that microRNA-101 regulates the downregulation of VE-cadherin and claudin-5 under hypoxic conditions, further implicating microRNA-mediated mechanisms in ischemia-induced BBB disruption [107].

### Parkinson’s Disease

Parkinson’s disease (PD) is a progressive neurodegenerative disorder primarily affecting motor function [108]. Disruption of the BBB in PD has been evidenced by significant elevations in albumin and IgG concentrations in the cerebrospinal fluid (CSF) of PD patients [109], as well as increased extravasation of erythrocytes, hemoglobin, and fibrin into the striatum [110]. Furthermore, immunofluorescence analyses have revealed a marked reduction in the TJ proteins ZO-1 and occludin within the substantia nigra, coinciding with increased IgG deposition in the parenchyma [58]. Notably, PD patients who underwent subthalamic deep brain stimulation (STN-DBS) exhibited attenuated BBB alterations, suggesting a preservation of microvascular integrity [111]. This observation provides a mechanistic basis for the therapeutic efficacy of STN-DBS, as reported in functional imaging studies.

Experimental models of PD further corroborate BBB dysfunction. In the 6-hydroxydopamine lesion model, BBB leakage was detected in the substantia nigra and striatum at both 10 and 34 days post-injection [112]. This was accompanied by significant reductions in the TJ proteins ZO-1 and claudin-5 [113]. Similarly, in the 1-methyl-4-phenyl-1,2,3,6-tetrahydropyridine (MPTP) mouse model of PD, increased BBB permeability to Evans blue and FITC-albumin was observed in the striatum but not in the hippocampus [114], indicating region-specific BBB impairment in PD pathophysiology.

### Traumatic Brain Injury

Traumatic brain injury (TBI) is a heterogeneous and dynamic pathology that varies in severity (mild, moderate, or severe) and classification (impact vs. non-impact, focal vs. diffuse). One of the hallmark pathophysiological features of TBI is the disruption of the BBB [84, 115–119]. The most severe consequence of TBI is intracranial hemorrhage, which is frequently observed in moderate to severe cases [120]. Similar to ischemic stroke models, BBB disruption in preclinical TBI models can exhibit monophasic or biphasic patterns, occurring within minutes to days post-injury. Notably, in a murine cerebral contusion model, BBB permeability to horseradish peroxidase (~44 kDa) persisted for up to 5 h, while permeability to smaller molecules (~0.3–10 kDa) was detectable for up to 4 days, indicating size-selective BBB leakage following TBI [121]. TJ proteins, including ZO-1, occludin, and claudin-5, are typically downregulated in the acute phase of TBI but may exhibit compensatory upregulation one to 2 weeks post-injury, coinciding with BBB recovery [122, 123]. In addition to paracellular disruption, TBI has also been implicated in increased transcellular permeability of the BBB [124]. Interestingly, even mild trauma can lead to quantifiable BBB dysfunction. In male C57Bl/6 mice, a study showed that moderate closed-skull TBI resulted in increased albumin extravasation at 24 h post-injury [124]. Multiple molecular mechanisms have been implicated in BBB disruption following TBI, including transforming growth factor-β (TGF-β) signaling, glutamate excitotoxicity, oxidative stress via reactive oxygen species, MMP activation, neuroinflammation, and VEGF signaling [125].

A frequently overlooked aspect of TBI is mild traumatic brain injury in military personnel exposed to repeated low-intensity blast overpressure (relBOP) from breaching barriers and operating heavy weaponry. A recent investigation has shown a significant upregulation of claudin-5 in the cortex, frontal cortex, and hippocampus following a single 6.5 psi blast exposure, though no clear pattern was identified regarding cumulative exposure effects. Additionally, TAR DNA-binding protein 43 (TDP-43) and glial fibrillary acidic protein (GFAP) exhibited mixed expression across various brain regions following blast exposure, suggesting potential neuropathological changes associated with relBOP-induced BBB dysfunction [126].

### Multiple Sclerosis

Multiple sclerosis (MS) is a chronic, progressive neuroinflammatory disorder characterized by immune-mediated damage to myelinated fibers, leading to demyelination, axonal degeneration, and lesion formation [127]. Disruption of the BBB is a critical event in MS pathogenesis, influencing lesion development, oligodendrocyte apoptosis, and neuronal injury [128, 129]. Gadolinium-enhanced magnetic resonance imaging (MRI) has demonstrated that BBB breakdown is among the earliest detectable events in forming new MS lesions. While vascular TJ alterations and serum protein extravasation are predominantly observed within MS lesions, they are also present in seemingly normal white and gray matter, suggesting a more extensive and systemic impairment of BBB integrity [130–133]. Structural abnormalities in TJs are frequently detected in active white matter lesions in both primary and secondary progressive MS, with occasional persistence in quiescent lesions [134]. Animals with experimental autoimmune encephalomyelitis (EAE), the most widely used in vivo model for MS, exhibit BBB dysfunction analogous to that observed in human MS, making this model valuable for investigating the underlying disease mechanisms despite some limitations. In EAE, as in human MS, there are selective alterations in TJ proteins, including a notable loss of claudin-3, while other key TJs such as ZO-1, occludin, and claudin-5 are also significantly disrupted, contributing to barrier breakdown and neuroinflammation [135, 136].

However, other studies reported that early EAE pathology induced a punctate distribution of claudin-5 while occludin underwent cytoplasmic translocation, disrupting TJ architecture [137, 138]. Additionally, relocalization of ZO-1 in EAE correlated with regions of inflammatory cell infiltration and preceded overt clinical manifestations [139]. Increased BBB permeability to Evans blue dye in EAE mice coincided with significant reductions in claudin-5, occludin, and ZO-1 expression, alterations that may be attenuated by resveratrol administration [140]. Kv1.3 channel blockade has similarly been shown to prevent BBB permeability changes and downregulation of TJ proteins in cerebellar white matter [141]. Notably, both EAE and MS patients exhibited significant reductions in claudin-11, further implicating TJ dysregulation in disease pathology [142]. Discrepancies in TJ alterations reported across studies may be attributed to variations in EAE models, time points analyzed, or differences in tissue sampling and detection methodologies.

BBB and BSCB disruption facilitate CNS infiltration of immune cells, including T cells, macrophages, and neutrophils, culminating in multifocal demyelination and neurodegeneration [143]. While the role of BBB dysfunction in MS is well established, BSCB compromise has received less attention despite mounting evidence implicating its involvement. Claudin-11, a key transmembrane protein maintaining BBB and BSCB integrity, is significantly downregulated in both MS patient tissue and EAE models, further linking barrier disruption to disease progression [142]. MRI-based investigations in EAE models reveal that BSCB disruption peaks in the early disease phase, diminishing as the disease progresses [144]. This finding aligns with evidence demonstrating that acute breakdown of the BBB occurs within a day of symptom onset, which correlates with a 4-day period of glial fibrillary protein+ myeloid cell infiltration into the CNS [145].

Notably, neutrophil recruitment to the lumbar spinal cord appears to mediate BSCB permeability to small tracer molecules. Targeted depletion of neutrophils using anti-lymphocyte antigen 6 complex locus G6D therapy delayed disease onset and reduced severity while simultaneously decreasing BSCB permeability, underscoring the contribution of neutrophils to BSCB dysfunction [145]. Additionally, activated microglia are implicated in barrier breakdown, further exacerbating MS pathology [146]. A recent study suggests a critical role for oligodendrocyte gap junction proteins in regulating BSCB integrity and inflammatory responses in MS. Connexin 32 and connexin 47 knockout (KO) mice exhibited exacerbated EAE pathology, with connexin 47 KO animals showing an earlier onset and increased disease severity. Upregulation of VCAM-1 expression occurred at multiple disease stages (7-, 12-, and 24-days post-induction) in connexin 47 KO EAE mice, reflecting dysregulated inflammatory signaling. Additionally, early-stage connexin 47 KO EAE mice displayed heightened T-cell infiltration, astrocyte activation, and abnormal TJ formation at the glia limitans, further compromising BSCB integrity. This suggests that connexin 47 deficiency potentiates inflammatory responses and accelerates barrier dysfunction, exacerbating disease progression. Further research is warranted to elucidate the precise role of oligodendrocytic connexin 47 in MS and EAE pathophysiology [147].

### Huntington’s Disease

Huntington’s disease (HD) is an autosomal dominant neurodegenerative disorder characterized by progressive motor dysfunction, cognitive decline, and psychiatric disturbances [148]. The pathological hallmark of HD includes intracellular aggregates of mutant huntingtin protein, which have been detected in the blood vessels of the caudate nucleus in HD patients, as well as in the brain tissue of R6/2 mice, a widely used preclinical model of HD [149]. Neuroimaging studies using gadolinium-diethylenetriamine pentaacetic acid (~550 Da) have observed increased BBB permeability in HD patients, correlating with postmortem findings of elevated fibrin deposition in the brain parenchyma. These vascular abnormalities were accompanied by a significant downregulation of critical TJ proteins, including occludin and claudin-5, suggesting that BBB dysfunction plays a role in disease progression [149]. In vitro models further support these findings, as brain endothelial cells derived from induced pluripotent stem cells (iPSCs) of HD patients exhibited aberrant claudin-5 localization and reduced transepithelial/transendothelial electrical resistance (TEER) values, indicative of impaired barrier integrity [150]. Preclinical studies using the R6/2 mouse model provide additional mechanistic insights into BBB dysfunction in HD. A significant reduction in occludin and claudin-5 expression, widening of the intercellular cleft at TJs, and increased BBB permeability via both transcellular and paracellular pathways have been observed [149]. Furthermore, age-dependent exacerbation of BBB impairment has been reported, with increased permeability to FITC-albumin, concomitant with progressive declines in claudin-5, occludin, and ZO-1 mRNA levels, as well as claudin-5 protein expression in the cortex and striatum [151]. These findings underscore the contribution of BBB dysfunction to HD pathophysiology and highlight potential therapeutic targets for mitigating neurovascular impairment in the disease.

### Neuropathic Pain

Neuropathic pain (NP) is a debilitating condition associated with dysfunction of the blood-spinal cord barrier (BSCB). Cardoso et al. have demonstrated that BSCB disruption contributes to NP pathogenesis by facilitating the infiltration of inflammatory mediators and immune cells into the spinal cord, thereby exacerbating nociceptive hypersensitivity [152]. The cyclophilin A (CypA)-MMP9 signaling pathway has been implicated in BSCB breakdown and NP development. A previous study revealed that inhibition of CypA significantly alleviated mechanical allodynia and thermal hyperalgesia, reduced BSCB permeability, and restored TJ protein levels [153]. The activation of the CypA-MMP9 axis induced microglial activation and inflammatory responses, leading to structural and functional impairment of the BSCB in a chronic constriction injury (CCI)–induced NP model. Elevated levels of proinflammatory cytokines were detected in CCI animals, further supporting the role of neuroinflammation in BSCB dysfunction and NP pathogenesis [153].

Peripheral nerve injury induces alterations in the spinal microenvironment that contribute to NP by promoting BSCB leakage, independent of spinal microglia activation. Monocyte chemoattractant protein-1 (MCP-1) has been identified as an endogenous trigger for BSCB disruption while circulating IL-1β further compromises barrier integrity [154]. Conversely, the anti-inflammatory cytokines TGF-β1 and interleukin-10 (IL-10) have been shown to restore BSCB integrity following nerve damage [155]. Notably, TGF-β1 preserved TJ proteins ZO-1 and occludin but failed to rescue caveolin-1, suggesting distinct regulatory mechanisms governing BSCB repair. The loss of TJ and caveolae-associated proteins following peripheral nerve injury facilitates the entry of inflammatory mediators and the recruitment of spinal blood-borne monocytes/macrophages, both of which play critical roles in NP development.

### Diabetic Retinopathy

Diabetic retinopathy (DR) is associated with dysregulation of the BRB, driven primarily by the upregulation of VEGF, which disrupts endothelial TJs [156]. Glial cell line-derived neurotrophic factor (GDNF), secreted by retinal astrocytes, plays an essential role in maintaining BRB integrity, with receptors for GDNF expressed on endothelial cells in the retina [157, 158], and across other tissues such as the BBB [159]. In DR, advanced glycation end-products (AGEs), products of hyperglycemia, interact with endothelial cells to stimulate VEGF production, which increases endothelial permeability by destabilizing TJs and promoting the degradation of GDNF, thus impairing barrier function [160].

In the early stages of DR, VEGF-induced alterations in the BRB contribute to increased leakage, particularly at the endothelial junctions. Retinoic acid receptor alpha (RAR-α) has been shown to attenuate VEGF expression in retinal astrocytes, thereby mitigating this permeability and stabilizing the BRB. RAR-α activation in this context results in a significant reduction of dye leakage from the BRB, reinforcing the notion that VEGF plays a pivotal role in the pathogenesis of early-stage DR [161]. As TJ dysfunction is a hallmark feature of DR, these observations suggest that therapeutic strategies aimed at preserving the integrity of the BRB during the early stages of the disease should focus on modulating astrocyte activity and reducing VEGF-mediated barrier disruption [162].

Additionally, n-butylidenephthalide (BP) has been shown to mitigate hyperglycemia-induced damage to the BRB in STZ-induced diabetic C57BL6 mice and ARPE-19 RPE cell cultures. BP treatment preserved TJ protein expression (ZO-1, claudin-1, occludin) and RPE65 expression, which are typically downregulated under hyperglycemic conditions. Notably, BP significantly upregulated these proteins in the RPE layer compared to STZ-treated controls, providing evidence for BP’s potential to preserve both RPE function and TJ integrity during diabetic retinal damage [163]. These findings underscore the potential of BP as a therapeutic agent for stabilizing the BRB and preventing early-stage diabetic retinal vascular dysfunction.

### Hypertension

The relationship between endothelial TJs and hypertension has been investigated for over four decades, revealing significant alterations in TJ integrity that contribute to vascular dysfunction. In spontaneously hypertensive rats (SHRs), a model of stroke-prone hypertension, a marked reduction in endothelial TJ expression precedes the onset of hypertension and is associated with an increased risk of BBB disruption and cerebrovascular events, such as stroke [164]. This suggests that TJ dysfunction is an early event in the pathophysiology of hypertension, potentially facilitating subsequent vascular damage.

In SHRs, the upregulation of JAM-A, a single transmembrane protein of TJs, has been observed throughout the body. JAM-A expression in the nucleus tractus solitarii has been shown to raise arterial pressure, implicating it as a key mediator of prohypertensive signaling within the brainstem [165]. Additionally, the loss or mislocalization of TJ proteins in hypertensive states results in TJ opening, which allows macrophages to infiltrate vascular tissues. This macrophage infiltration, in turn, activates MMP-9, which is implicated in the formation of cerebral aneurysms [166]. These findings underscore the role of endothelial TJs in both the development and progression of hypertension, highlighting their involvement in regulating vascular permeability and inflammatory responses that contribute to cerebrovascular complications.

### Brain Tumors

The integrity and permeability of the BBB are significantly compromised in the presence of brain tumors, leading to the formation of a heterogeneous blood-tumor barrier (BTB) with distinct characteristics, including non-uniform permeability and active molecular efflux [167]. Approximately 30% of brain tumors are metastatic, originating from melanomas, breast cancer, and lung cancer [168]. The disruption of inter-endothelial TJs has been well-documented in human metastatic adenocarcinomas and gliomas [169, 170], further contributing to BBB dysfunction. ZO-1 is a scaffold protein that cross-links and anchors the TJ strand proteins to the cell cytoskeleton.

Despite the downregulation of these TJ proteins in glioblastoma multiforme (GBM) patients, ZO-1 expression remains unchanged in the brain microvessels [169]. This disruption in TJ structure contributes to increased BBB permeability, which facilitates the development of cerebral edema, partly driven by VEGF and pro-inflammatory cytokines secreted by tumor cells [171]. Furthermore, these cytokines are implicated in the opening of the BBB and are associated with elevated expression of aquaporin-4 (AQP4) in astrocytomas and metastatic adenocarcinomas [172]. Mice deficient in AQP4 have been shown to survive longer than wild-type counterparts in in vivo brain edema models, highlighting its potential role in exacerbating BBB dysfunction [173].

The breakdown of the BBB can enable metastatic tumor cells to infiltrate the brain parenchyma despite the brain’s inherent defense mechanisms against malignant cell entry. The structural integrity of the BBB and BTB varies depending on the type of tumor and its metastatic lesions [167]. For instance, four distinct genetic subtypes of medulloblastoma exhibit different levels of fenestration in endothelial cells, which can influence treatment efficacy across the BTB [174]. Moreover, the BBB characteristics in brain metastases originating from breast cancer differ between subtypes; human epidermal growth factor receptor 2-positive metastases, for example, show higher expression of GLUT-1 and breast cancer resistance protein (BCRP) compared to other subtypes [175]. These variations in BTB permeability are further corroborated by pre-clinical studies, which note that the tumor core is generally more permeable than the peritumoral area, affecting therapeutic distribution. For example, in the intracranial GBM8401 glioma model, doxorubicin-containing liposomes exhibit greater distribution within the tumor than in surrounding brain tissue [176].

Recent studies have also explored the role of competing endogenous ribonucleic acids (ceRNAs) in regulating claudin-5 expression in brain vascular endothelial cells. Dysregulated ceRNAs, which alter claudin-5 levels, may contribute to the formation and progression of brain metastasis [177]. These findings suggest that targeting ceRNA-mediated alterations in TJ protein expression may provide novel therapeutic strategies for preventing or mitigating brain metastasis.

### HIV-1-Associated Encephalitis

Human immunodeficiency virus (HIV) infection can lead to neurological conditions, such as dementia and HIV-1-associated encephalitis (HIVE), when the virus penetrates the CNS. Studies examining HIV-1-associated encephalitis brain tissue using immunohistochemistry have shown that occludin and ZO-1 immunoreactivity is fragmented or completely absent in vessels of the subcortical white matter, basal ganglia, and, to a lesser extent, cortical gray matter. This breakdown of TJ proteins is closely associated with BBB leakage, which coincides with the presence of activated HIV-1-infected brain macrophages and fibrinogen leakage [178].

Similar alterations in TJ proteins have been observed in the CNS microvessels of individuals with HIV-1-associated dementia, where disrupted ZO-1 and occludin immunoreactivity were found in microvessels encircled by CD68-positive macrophages [179]. HIV-positive monocytes are believed to directly impact the expression and functionality of TJ proteins by producing metalloproteases, reactive oxygen species, and pro-inflammatory cytokines [180]. Persistent oxidative stress, driven by excess free radicals, is a characteristic feature of HIV-1 infection and endothelial dysfunction [181]. HIV-1-positive individuals exhibit lower levels of circulating antioxidants, such as vitamin C, cysteine, and glutathione, and higher plasma levels of hydroperoxides, indicating increased free radical production and lipid peroxidation [182]. Viral proteins, particularly gp120, have been shown to affect BBB permeability in vivo and disrupt TJ integrity in in vitro BBB models by inducing proteasome-dependent degradation of ZO-1 and ZO-2 and the loss of occludin [183–185].

Interestingly, while the exposure of brain microvascular endothelial cells to the HIV-1 Tat protein leads to decreased expression of claudin-1, claudin-5, and ZO-2, the expression of ZO-1 and occludin remains unchanged [186]. In contrast, studies have shown that intrahippocampal injection of Tat in mice results in lower expression of claudin-5, indicating Tat-induced alterations in TJ protein expression in CNS microvessels [187]. This finding contradicts a study in which intravenous Tat treatment in mice led to increased expression of cyclooxygenase-2 (COX-2) and decreased expression of ZO-1 and occludin [188]. ZO-1 alterations in this context were associated with changes in the extracellular signal-regulated kinase (ERK)1/2 redox signaling pathway. When pretreated with the antioxidant N-acetylcysteine, these ZO-1 and ERK1/2 activation changes were significantly attenuated [188]. Tat-induced downregulation of claudin-5 has been linked to the activation of redox-regulated signaling pathways, including Ras/ERK1/2 and AKT/BK/NF-κB [189]. Furthermore, exposure to increasing doses of Tat in vitro decreased glutathione (GSH) levels, increased oxidative stress, and activated redox-responsive transcription factors like NF-κB and activator protein-1 in brain microvascular endothelial cells [190]. In vivo studies have also shown that injecting gp120 into the caudate-putamen of rats induces oxidative stress and BBB leakiness, with elevated MMP-2 and MMP-9 [191]. Furthermore, studies examining the diapedesis of HIV-1-infected monocytes across an in vitro BBB model have demonstrated increased Rho kinase-mediated phosphorylation of occludin and claudin-5, linking immune cell migration across the BBB to TJ alterations [192].

Tat exposure has also been shown to directly influence the expression of adhesion molecules, such as VCAM-1 and ICAM-1, as well as inflammatory mediators like monocyte chemoattractant protein-1 (MCP-1) and -TNF-α [193]. These mediators promote monocyte infiltration into the brain. Furthermore, Tat activation of brain microvascular endothelial cells leads to the production of E-selectin, facilitating leukocyte adhesion to the BBB. In mice, Tat injection into the hippocampus results in endothelial production of MCP-1, which is regulated by redox-sensitive transcription factors NF-κB and activator protein 1. These findings suggest that Tat directly induces BBB breakdown through redox-related inflammatory responses, contributing to the pathogenesis of HIV-associated neurological disorders [190].

### Sickle Cell Disease

Sickle cell disease (SCD) profoundly affects the CNS barrier, endothelial barrier function, and TJ proteins, contributing to neurological complications and vascular instability [194]. The pathophysiology of SCD is complex, involving endothelial activation, BBB disruption, and altered cerebral blood flow dynamics, all driven by chronic inflammation, oxidative stress, and recurrent vaso-occlusive events [195, 196]. Recent studies have shown that BBB permeability is elevated in SCD patients compared to healthy controls, with this increased permeability being associated with stroke and microstructural injury [197]. Endothelial dysfunction, a hallmark of SCD, plays a critical role in vascular instability and BBB disruption. SCD patients exhibit altered cerebral blood flow and decreased cerebrovascular reserve, indicating compromised endothelial function [198]. Further investigation has highlighted the critical role of mast cells in BBB disruption in SCD. These cells induce endoplasmic reticulum (ER) stress in endothelial cells, contributing to barrier dysfunction [199]. Additionally, a study found that sickle cell mice exhibited impaired pulmonary endothelial barrier function, showing increased susceptibility to lipopolysaccharide (LPS)-induced toxicity compared to controls. The treatment with β-nicotinamide adenine dinucleotide (β-NAD) improved basal endothelial barrier function and protected against LPS toxicity in sickle cell mice [200].

Circulating extracellular vesicles (EVs) derived from SCD patients, especially during acute chest syndrome (ACS) episodes, have been shown to disrupt endothelial barrier function. These vesicles contribute to the dysregulation of junction proteins, which are critical for maintaining the integrity of the CNS barrier. Gap junctions, composed of connexin proteins such as Connexin 43, appear particularly vulnerable to disruption by SCD-derived EVs. This disruption occurs earlier and more severely than alterations in TJs and AJs, suggesting a hierarchical model of junction protein dysregulation in SCD [201].

### Spinal Cord Injury

Spinal cord injury (SCI) leads to significant disruption of the BSCB, exacerbating inflammation, oxidative stress, edema, bleeding, and neuronal damage [202–204]. The breach of this barrier can be primarily attributed to the breakdown of TJs that typically link vascular endothelial cells. Following SCI, blood-derived macrophages infiltrate the spinal cord, congregate in the injury core, and polarize toward M1/M2 phenotypes, influencing the BSCB’s integrity. Luo et al. showed that claudin-5 and ZO-1 expression in vascular endothelial cells within the injury core significantly decreases compared to that in normal spinal cord tissue. Furthermore, this alteration persisted for 7 to 28 days post-injury (dpi). Macrophage accumulation in the injury core was concomitant with BSCB leakage, as demonstrated by FITC-dextran perfusion. Endothelial dysfunction, especially in the form of altered TJ protein expression, plays a central role in BSCB disruption following SCI. Macrophage infiltration and the associated inflammatory response further exacerbate the breakdown of junctional integrity, thus amplifying vascular permeability. Research has suggested that early intervention to modulate macrophage activity can aid in restoring TJs and reduce the leakage of the BSCB [205].

Additionally, the cellular signaling mechanisms post-SCI further complicate the repair of the BSCB. After injury, endothelial cells participate in clearing myelin debris, which can induce additional damage to TJs and gap junctions. The uptake of myelin debris by endothelial cells stimulates the activation of signaling pathways, such as PI3K/AKT and ERK, which contribute to endothelial cell migration and the endothelial-to-mesenchymal transition. This process increases endothelial permeability and exacerbates BSCB dysfunction, hindering recovery and healing [206].

Furthermore, alterations in the expression of junctional proteins like occludin, claudin-5, and ZO-1 significantly affect barrier integrity. The activation of matrix metalloproteinases, particularly MMP-9, plays a pivotal role in TJ degradation, thus influencing the progression of SCI and the severity of BSCB leakage [207]. In the chronic phase of SCI, the inflammatory response continues to drive changes in endothelial cell behavior and junctional protein expression, impairing the recovery of the BSCB. The role of caveolin-1 in maintaining BSCB integrity has been recognized, as it regulates both barrier permeability and the expression of junction proteins. In SCI models, the expression of caveolin-1, along with other junctional proteins, is compromised, but treatments such as basic FGF have been shown to modulate this pathway and improve the recovery of BSCB function [208]. However, the primary challenge lies in understanding how these mechanisms are integrated and how the barrier’s recovery can be optimally managed to limit secondary neuronal injury.

### Amyotrophic Lateral Sclerosis

Amyotrophic lateral sclerosis (ALS), a fatal neurodegenerative disease characterized by the rapid degeneration of motor neurons in the brain and spinal cord, has been associated with significant alterations to the BBB and BSCB [209]. While these vascular changes have not been as extensively studied as those in other neurodegenerative conditions, accumulating evidence suggests that they play a pivotal role in the pathophysiology of ALS [210, 211]. The disease course is marked by progressive motor dysfunction and typically results in death within 2 to 4 years following symptom onset [212]. In ALS, these TJ proteins are significantly reduced [213], leading to endothelial dysfunction and compromised barrier permeability.

In transgenic mouse models with ALS-linked SOD1 mutations, a marked decrease in the expression of TJ proteins ZO-1, occludin, and claudin-5 was observed, reflecting impaired BBB and BSCB function [214]. This reduction is not restricted to animal models; human postmortem spinal cord tissues have also shown similar deficits in TJ protein expression, particularly in the lumbar spinal cord regions, which are heavily affected by ALS pathology [215]. In ALS patients, postmortem analysis of spinal cord tissue has revealed a marked reduction in occludin levels and diminished perivascular collagen type IV and astrocytic end-feet encircling endothelial cells [216]. These changes collectively contribute to a dysfunctional NVU, facilitating BBB and BSCB breakdown, subsequently allowing potentially neurotoxic substances from the periphery to enter the CNS parenchyma, exacerbating neurodegeneration [217]. The pathological changes in ALS extend beyond TJ protein degradation, including a reduction in key endothelial cell markers such as GLUT-1 and cluster of differentiation-146. These proteins are critical for the maintenance of endothelial integrity and the proper function of the BBB. In ALS, the expression of these molecules is markedly decreased, further contributing to endothelial dysfunction and vascular instability [217]. In SOD1-mutant mouse models, a reduction in blood flow to the cervical and lumbar spinal cord is observed before symptom onset, with a reduction of 30% to 45% in spinal cord blood flow, further indicating a pre-symptomatic alteration of vascular dynamics [213].

ALS is associated with profound changes in the vasculature and NVU at molecular and ultrastructural levels. RNA sequencing and immunolabeling data from SOD1G93A mutant mice have revealed a significant loss of capillary density and TJ integrity in the lumbar spinal cord that precedes observable neuromuscular junction denervation. Ultrastructural analysis further identifies string vessels, increased pericytes, and a thickened basement membrane, indicating early vascular remodeling in ALS [218–220]. These vascular alterations occur before the clinical onset of motor neuron degeneration, suggesting that changes to the NVU may play a critical role in ALS pathogenesis and may be involved in early disease onset and progression.

### Spinal Cord Carcinoma

Spinal cord carcinoma is a rare entity, representing only 10–15% of CNS tumors, with the majority (80–85%) occurring in the brain [221]. While there is no direct evidence linking tumor mass expansion to disruption of the BSCB, perturbations in BSCB integrity have been observed following radiation therapy and other anti-tumor treatments. Both acute and chronic radiation exposure have been shown to induce structural alterations in endothelial cells and TJ proteins within the BBB [222]. Experimental studies investigating BSCB disruption in rat spinal cords following irradiation have demonstrated that serum albumin leakage occurs 24 h post-irradiation, which correlated with heightened ICAM-1 expression on both endothelial and glial cells. While elevated ICAM-1 expression is not directly attributable to radiation exposure, it is likely a consequence of the activation of downstream signaling pathways, including those involving growth factors, cytokines, and transcription factors induced by radiation. Following CNS radiation injury, hypoxia has been shown to upregulate VEGF in astrocytes, contributing to BSCB disruption, as evidenced by albumin extravasation in rat spinal cords [223]. Notably, BSCB disruption was observed in areas of the spinal cord undergoing tissue damage, both before and after the onset of necrosis. This disruption was closely associated with the expression of genes responsive to hypoxic and regional hypoxic conditions. VEGF has been implicated in forming fenestrations in capillary endothelial cells and modulating occludin expression and tight junction assembly, thereby contributing to the compromise of BSCB integrity [224–226].

## Therapeutic Approaches to Restore CNS Tight Junctions

Therapeutic strategies aimed at restoring the integrity of the BBB and BSCB primarily focus on reinforcing their structural and functional stability, which is essential for maintaining CNS homeostasis [227]. These approaches include the use of pharmacological agents that modulate TJ proteins between endothelial cells to re-establish barrier function, as well as therapies that attenuate inflammatory responses and oxidative stress, two key factors that compromise barrier integrity. In addition, gene therapy is being investigated as a means to deliver targeted molecular interventions directly to sites of injury [228]. These interventions are designed to promote barrier repair, reduce neuroinflammation, and enhance functional recovery in neurodegenerative and neurovascular disorders [229, 230]. A detailed overview of therapeutic strategies directed at barrier restoration is provided in Table 1.

**Table 1:** Therapeutic strategies targeting CNS TJs in regulating BBB/BSCB permeability

Therapeutic approach	Mechanism	Effect on barrier
Growth factors	• FGF1 drives PRDX1-dependent autophagy and anti-reactive oxygen species activity and protects endothelial junctional integrity [231, 232] • PDGF-D/B → PDGFRβ signaling control junctional equilibrium and pericyte coverage [234] • TGF-β from pericytes upregulates TJ expression [235] • Ang-1/Tie2 stabilizes junctions [236] • GDNF via PI3K/AKT and MAPK/ERK activation upregulates VE-cadherin and claudin-5 [237]	Strengthened TJs and restored BBB/BSCB integrity
MicroRNA-based approaches	• Inhibition of miR-15a/16-1 upregulates claudin-5 [239, 240] • Suppression of miR-155 upregulates occludin and claudin-5 [241] • Inhibition of miR-501-3p restores ZO-1 [242, 243] • Inhibition of miR-29c-5p, miR-132/212 lowers endothelial permeability [244]	TJ proteins maintenance and reduced endothelial permeability
Pharmacological agents	• Fasudil-mediated ROCK inhibition prevents stress fibers and maintains TJ proteins [245] • Statins conserve TJs and reduce inflammation [246, 247] • Edaravone scavenges free radicals and preserves pericytes [248] • Metformin/PPARα agonists increase claudin-5/occludin [249, 250]	Preserved TJ distribution and strengthened BBB
Extracellular vesicles	• MSC-EVs modulate microglial responses and lower inflammation [251–253] • MSC exosomes from bone marrow regulate MMPs, activate Akt, and reduce endoplasmic reticulum stress [255] • Exosomes from platelet-rich plasma stabilize BSCB [256, 257]	Restored BSCB/BBB
MMP inhibitors	• Broad MMP inhibition prevents TJ proteolysis [258, 259] • Minocycline suppresses MMPs and preserves occludin/claudin-5 [260] • MMP-9 neutralizing antibodies block TJ cleavage [261] • Resveratrol/EGCG inhibit MMPs and upregulate TJ proteins [262–264]	Preserved ZO-1, claudin-5, occluding and reduced BBB leakage
Stem cell–based therapies	• MSCs/NSCs restore ZO-1, occludin, and claudin-5 via immunomodulation, angiogenesis, and neurogenesis promotion and strengthen endothelial–pericyte interactions [265–267, 271–273] • MSCs reduce proinflammatory cytokines (TNF-α and IL-1β) and upregulate anti-inflammatory factors [268–270] • EPCs differentiate into endothelial cells and enhance the function of blood vessel lining [266, 274]	Enhanced TJ expression and restored BBB/BSCB integrity

### Growth Factor-Based Approaches

Fibroblast growth factor 1 (FGF1) promotes post-SCI recovery by reducing tissue loss, preserving neuronal viability, and facilitating axonal regeneration. Mechanistically, FGF1 enhances autophagy and antioxidant responses through a peroxiredoxin 1 (PRDX1)-dependent pathway, thereby indirectly protecting the integrity of endothelial tight junctions [231, 232]. These effects are mediated by increased autophagy and upregulated peroxiredoxin 1 expression, which together enhance anti-reactive oxygen species activity. Despite its therapeutic potential, the clinical use of FGF1 remains limited due to its instability and poor permeability across the BSCB. To overcome these limitations, acidic FGF-loaded hydrogels have been developed, enabling sustained intralesional delivery in preclinical models of SCI [233].

Other growth factors, including platelet-derived growth factor (PDGF) and TGF-β, contribute to the restoration of BBB integrity by modulating pericyte-endothelial interactions and upregulating TJ protein expression, thereby consolidating barrier function [26]. Specifically, PDGF-D and PDGF-B/PDGF receptor β signaling pathways regulate junctional equilibrium and promote appropriate pericyte coverage, restoring barrier function in vivo [234]. Similarly, pericyte-derived TGF-β enhances TJ expression and supports endothelial quiescence [235].

In addition, pericyte- and astrocyte-mediated angiopoietin-1/Tie2 signaling maintains endothelial junctional equilibrium and counterbalances angiopoietin-2-driven vascular leakage, thereby strengthening the barrier within the CNS endothelium [236]. Moreover, neuron-derived glial cell line-derived neurotrophic factor (GDNF) upregulates VE-cadherin and claudin-5 expression in brain endothelial cells via PI3K/AKT/Ets-1 and MAPK/ERK/Ets-1 signaling pathways, as demonstrated in brain-specific GDNF-silencing mouse models [237].

### MicroRNA-Based Approaches

MicroRNAs (miRNAs) have recently attracted considerable attention as regulators of endothelial function and as potential therapeutic tools for restoring BBB integrity [238]. Rather than acting as isolated effectors, miRNAs orchestrate complex signaling networks that influence TJ stability and vascular permeability, thereby offering new avenues for barrier repair in neurological disease.

Among the best-characterized examples is the miR-15a/16-1 cluster, whose inhibition in endothelial cells enhances claudin-5 expression, reduces vascular leakage, and confers resistance to ischemic injury [239, 240]. Similarly, miR-155 has emerged as a central mediator of inflammatory barrier disruption. In models of MS, targeted suppression of miR-155 preserved occludin and claudin-5 expression, stabilized BBB function, and translated into a 40% reduction in disease severity compared with controls [241]. These findings underscore the therapeutic potential of miRNA modulation not only in acute injury but also in chronic neuroinflammatory conditions.

Other studies have extended this paradigm to vascular cognitive impairment, where TNF-α-induced miR-501-3p was shown to downregulate ZO-1 and compromise junctional integrity. Inhibition of miR-501-3p restored TJ architecture and improved cognitive outcomes, highlighting the possibility of targeting miRNAs to address both vascular and functional decline [242, 243]. Beyond these individual examples, miRNAs such as miR-29c-5p and miR-132/212 also appear to play protective roles by reducing endothelial permeability and reinforcing junctional proteins under ischemic stress [244].

### Pharmacological Approaches

A diverse range of pharmacological agents, both established drugs and novel compounds, has demonstrated potential for restoring BBB integrity. These agents act through complementary mechanisms, targeting cytoskeletal dynamics, inflammatory pathways, oxidative stress, and metabolic regulation to stabilize TJ complexes.

Fasudil, a Rho kinase inhibitor, is studied to prevent stress fiber formation and preserve the distribution of TJ proteins such as claudin-5 and occludin following ischemic stroke. By maintaining junctional integrity, fasudil not only reduced infarct volume by nearly 30% but also improved neurological recovery, underscoring the therapeutic importance of modulating cytoskeletal tension [245]. Similarly, statins such as simvastatin extend beyond their lipid-lowering effects to exert pleiotropic benefits on the BBB. Through conservation of TJ proteins, attenuation of inflammation, and protection against ischemic insults, statins have consistently been associated with improved neurological outcomes [246, 247].

Other pharmacological strategies focus on mitigating oxidative stress, a key driver of barrier breakdown. Free-radical scavengers such as edaravone, which has been repurposed as a cytoprotective agent, limit TJ disruption and pericyte loss in the context of reperfusion injury [248]. Complementing this approach, metabolic modulators including metformin and peroxisome proliferator-activated receptor-α (PPARα) agonists have been investigated for their ability to stimulate TJ proteins such as claudin-5 and occludin, thereby reducing barrier permeability and reinforcing vascular stability [249, 250].

### Extracellular Vesicles

EVs, particularly those derived from mesenchymal stromal cells (MSCs), have emerged as promising therapeutic agents for restoring barrier integrity after CNS injury [251]. By modulating intercellular communication, these vesicles influence inflammatory and reparative pathways that are critical for BBB and BSCB stabilization. Furthermore, they serve as vehicles for the delivery of protective microRNAs and proteins, which in turn enhance TJ protein expression and barrier resilience [252, 253].

In experimental SCI, MSC-derived EVs have been shown to reprogram microglial responses, thereby reducing neuroinflammation and promoting functional recovery, as demonstrated by improved mechanical sensitivity thresholds in treated animals [251, 254]. Extending these findings, bone marrow MSC-derived exosomes have demonstrated multifaceted mechanisms of action in BSCB repair. They regulate MMP activity, activate the Akt pathway, and mitigate endoplasmic reticulum stress mechanisms that converge to promote vascular repair and neurological recovery [255]. Similarly, exosomes obtained from platelet-rich plasma represent a novel therapeutic approach, stabilizing BSCB function and alleviating neuroinflammation to enhance post-injury outcomes [203]. Importantly, EVs amplify their actions by transferring bioactive molecules that directly influence gene expression in recipient cells [256, 257].

### Matrix Metalloproteinases Inhibitors

MMPs are key drivers of BBB disruption through proteolytic cleavage of basement membrane and TJ proteins, including ZO-1, claudin-5, and occludin, thereby serving as critical mediators of CNS. Their activity is strongly implicated in barrier breakdown following trauma, stroke, and neuroinflammation, positioning MMPs as key mediators of CNS injury and attractive therapeutic targets [101, 258].

Early studies with broad-spectrum MMP inhibitors, such as batimastat-94, demonstrated improved barrier integrity and reduced hemorrhagic complications by limiting proteolytic TJ degradation in preclinical models of ischemia and inflammation [259]. While the clinical translation of such agents has been limited due to off-target effects, these findings underscored the therapeutic value of blocking MMP activity. In parallel, repurposed agents like minocycline have gained attention for their ability to suppress MMP expression after CNS injury. Minocycline preserves occludin and claudin-5 localization, decreases edema, and mitigates BBB leakage in vivo, providing a clinically relevant example of MMP modulation [260].

More selective approaches, such as neutralizing antibodies directed against MMP-9, further highlight the promise of precision inhibition. These strategies attenuate TJ proteolysis and significantly reduce BBB disruption in models of stroke [261, 262]. Beyond synthetic inhibitors, naturally derived compounds including resveratrol and epigallocatechin gallate (EGCG) have emerged as nutraceutical candidates with MMP-inhibitory, anti-inflammatory, and antioxidant properties. By upregulating occludin and claudin-5 expression, these agents contribute to barrier stabilization and repair [263, 264].

### Stem Cell-Based Therapies

Stem cell-based approaches, with their vigorous advantages, have gained increasing recognition as potential strategies to restore BBB and BSCB integrity in the context of traumatic injury and stroke [265]. In the past decades, several cell types including iPSCs, MSCs, neural stem cells (NSCs), embryonic stem cells (ESCs), endothelial progenitor cells (EPCs), and some neural stem cell lines have been evaluated as implicit cell therapy for ischemic stroke [266]. The therapeutic efficacy of stem cell interventions exerts broad immunomodulatory effects, stimulates angiogenesis, promotes neurogenesis, and strengthens endothelial–pericyte interactions, all of which contribute to vascular stabilization [267]. NSCs or induced neural stem cells (iNSCs) have been shown to reinforce TJ proteins, including ZO-1, occludin, and claudin-5, thereby reducing barrier leakage and mitigating secondary injury [268–270]. MSCs hamper proinflammatory cytokines such as TNF-α and IL-1β while upregulating anti-inflammatory factors that are critical for repairing barrier integrity [268]. Preclinical studies in models of TBI and stroke demonstrate that MSCs effectively restore TJ integrity and reduce vascular permeability, underscoring their potential as scalable and clinically translatable therapeutics [271, 272]. MSCs isolated from bone marrow, adipose tissue, and umbilical cord have displayed therapeutic efficacy in animal models via secretion of paracrine factors that stabilize the endothelium in TBI [273]. On the other hand, EPCs play a key role in restoring BBB integrity through differentiating into endothelial cells, enhancing the potency of blood vessel lining. In addition, EPCs upregulate angiogenesis, which aids the overall tissue repair [274].

Nevertheless, poor graft survival, heterogeneity among cell populations, risks of tumorigenicity, and variability in delivery methods continue to hinder clinical translation. Future strategies emphasizing standardized protocols, engineered cell products, and adjunctive cell-free approaches may provide safer and more effective avenues for restoring BBB and BSCB integrity.

## Tight Junctions as Biomarkers of BBB Integrity

The identification of reliable biomarkers that reflect BBB integrity is crucial for advancing both mechanistic understanding and diagnostic approaches in neurological and psychiatric disorders [275]. TJ proteins have emerged as particularly promising candidates because of their central role in maintaining endothelial barrier properties and their dynamic regulation under pathological conditions [276]. Alterations in TJ protein expression, localization, and post-translational modifications provide valuable insight into BBB dysfunction and directly link molecular changes with disease onset and progression [51]. Disruption of TJ proteins is now recognized as a significant factor across a spectrum of neurological and psychiatric conditions [277]. Recent studies have emphasized the diagnostic potential of key TJ proteins, including ZO-1, occludin, and claudin-5, for assessing BBB integrity in diverse disease contexts [278]. Because these proteins are fundamental to maintaining selective BBB permeability, their dysregulation has been consistently associated with CNS pathology [278, 279].

In MS, aberrant TJ regulation, including altered expression and phosphorylation of claudin-5 and occludin, contributes to barrier dysfunction and facilitates immune cell infiltration, which drives neuroinflammation and disease progression [280]. In AD and PD, early BBB disruption coincides with reduced TJ protein expression, preceding cognitive decline and correlating with oxidative stress and neuroinflammation [281]. In untreated HIV infection, diminished TJ protein levels in CSF are strongly correlated with neuronal damage and cognitive impairment [282]. Following ischemic injury, degradation of claudin-5 and occludin by MMPs leads to increased permeability and exacerbated neuronal loss [279, 283]. Similarly, in TBI, elevated levels of MMP-3 are linked to the disintegration of TJ proteins, promoting BBB disruption, edema formation, and aggravated neuronal damage, thereby underscoring the potential of TJ proteins as indicators of BBB integrity post-injury [284]. In neonatal models of hypoxic–ischemic injury, altered TJ protein levels have been detected in CSF and plasma, suggesting their potential as biomarkers for assessing BBB function and providing insight into early brain injury [285]. Furthermore, emerging evidence indicates that TJ protein alterations may also contribute to BBB dysfunction in psychiatric conditions such as bipolar disorder and schizophrenia, where they are associated with cognitive impairments and neuroinflammation [286].

These findings establish TJ proteins as critical biomarkers of BBB integrity across a wide range of neurological and psychiatric disorders. Their measurable alterations in CSF, plasma, or brain tissue not only reflect barrier dysfunction but also parallel key pathological mechanisms such as oxidative stress, neuroinflammation, and neuronal damage. By providing accessible and dynamic readouts of barrier status, TJ proteins hold promise for improving early diagnosis, monitoring disease progression, and guiding the development of targeted therapeutic strategies.

## Conclusions and Future Perspectives

TJs play a pivotal role in maintaining the integrity of BBB and BSCB, establishing the stabilization of the CNS from noxious substances and pathogens while governing molecular trafficking. The dynamic regulation of TJs, mediated by various signaling pathways, is vital for their function in healthy conditions. Dysregulation of these tight junctions contributes to a wide range of neurological disorders, including neurodegenerative diseases and neuroinflammation, thus underscoring their importance in CNS homeostasis. Impending research should be directed at elucidating the definite molecular mechanisms that control tight junction dynamics in both health and disease. Advancing our understanding of how signaling pathways impact TJ assembly, disassembly, and permeability could accelerate novel therapeutic approaches aimed at restoring barrier integrity in conditions where it is compromised. Emerging therapies may entail the advancement of molecules that can selectively stabilize TJs to lower permeability in neuroinflammatory conditions. The conjunction of TJ biology and permeability stabilization approaches is a promising frontier that could revolutionize the treatment of CNS disorders. Persistent investigations of these avenues will be crucial to overcoming current barriers in both basic science and clinical applications.

## Data Availability

No datasets were generated or analysed during the current study.

## Abbreviations

ACS: Acute chest syndrome
AD: Alzheimer’s disease
AF-6: Afadin-6
AGEs: Advanced glycation end-products
AJs: Adherens junctions
AKT: Protein kinase B
ALS: Amyotrophic lateral sclerosis
APP: Amyloid precursor protein
AQP-4: Aquaporin 4
ARPE-19: Retinal pigment epithelia cell line
ATP: Adenosine triphosphate
Aβ: Amyloid beta
BBB: Blood-brain barrier
BCRP: Breast cancer resistance protein
bFGF: Basic fibroblast growth factor
BRB: Blood retinal barrier
BSCB: Blood-spinal cord barrier
BTB: Blood tumor barrier
β-NAD: Beta-nicotinamide adenine dinucleotide
CAMs: Cell adhesion molecules
Ca2^+^: Extracellular calcium
CAR: Coxsackievirus and adenovirus receptor
CCI: Chronic constriction injury
CCL2: C-C motif chemokine ligand 2
ceRNA: Competing endogenous ribonucleic acid
CNS: Central nervous system
CSF: Cerebrospinal fluid
CypA: Cyclophilin A
Dpi: Days post injury
EAE: Experimental autoimmune encephalomyelitis
EGCG: Epigallocatechin gallate
EPCs: Endothelial progenitor cells
ERK1/2: Extracellular signal-regulated kinases
ESAM: Endothelial cell-selective adhesion molecule
ESCs: Embryonic stem cells
EVs: Extracellular vesicles
FGF: Fibroblast growth factor
FITC: Fluorescein isothiocyanate
GBD: Global burden of disease
GBM: Glioblastoma multiforme
GD-DTPA: Gadolinium-diethylenetriamine pentaacetic acid
GDNF: Glial cell line-derived neurotrophic factor
GFAP: Glial fibrillary acidic protein
GLUT: Glucose transporters
Gp120: Envelope glycoprotein
GSH: Glutathione
HD: Huntington’s disease
HIV: Human immunodeficiency virus
HIVE: HIV encephalitis
ICAM-1: Intercellular adhesion molecule
IgG: Immunoglobulin
IL: Interleukin
IL-1β: Interleukin-1 beta
iNSCs: Induced neural stem cells
iPSCs: Induced pluripotent stem cells
JAM-L: Junctional adhesion molecule-like
JAMs: Junctional adhesion molecules
KO: Knock out
LPS: Lipopolysaccharide
LSR: Lipolysis-stimulated lipoprotein receptor
MAGUK: Membrane-associated guanylate kinase
MAPK: Mitogen-activated protein kinase
MARVEL: Myelin and lymphocyte and related proteins for vesicle trafficking and membrane link
MCAO: Middle cerebral artery occlusion
MCP-1: Monocyte chemoattractant protein-1
miRNA: Micro RNA
MLCK: Myosin light chain kinase
MMP: Matrix metalloproteinases
MPTP: 1-Methyl-4-phenyl-1,2,3,6-tetrahydropyridine
MRI: Magnetic resonance imaging
MS: Multiple sclerosis
MSCs: Mesenchymal stromal cells
nBP: n-Butylidenephthalide
L-NAME: *N*^G^-nitro-^l^-arginine methyl ester
NF-κB: Nuclear factor kappa-light-chain-enhancer of activated B cells
NP: Neuropathic pain
NSCs: Neural stem cells
NVU: Neurovascular unit
PDGF: Platelet-derived growth factor
PECAM-1: Platelet/endothelial cell adhesion molecule-1
PRDX1: Peroxiredoxin 1
PI3K: Phosphatidylinositol 3-kinase
PKC: Protein kinase C
PPARα: Peroxisome proliferator-activated receptor-α
RAR-α: Retinoic acid receptor alpha
relBOP: Repeated low-intensity blast overpressure
RNAi: RNA interference
RPE: Retinal pigment epithelium
SCD: Sickle cell disease
SCI: Spinal cord injury
SE: Status epilepticus
Ser: Serine
SHRs: Spontaneously hypertensive rats
SOD1: Superoxide dismutase 1
STN-DBS: Subthalamic deep brain stimulation
STZ: Streptozotocin
Tat: Transactivator of transcription
TBI: Traumatic brain injury
TDP43: TAR DNA-binding protein 43
TEER: Transepithelial/transendothelial electrical resistance
TEMPO: 4-Hydroxy-2,2,6,6-tetramethylpiperidine-N-oxyl
TGF-β: Transforming growth factor beta
Thr: Threonine
TJ: Tight junction
TNFα: Tumor necrosis factor-alpha
TRPM7: Transient receptor potential melastatin 7
Tyr: Tyrosine
VCAM-1: Vascular cell adhesion molecule-1
VE-Cadherin: Vascular endothelial cadherin
VEGF: Vascular endothelial growth factor
WHO: World Health Organization
ZO-1: Zonula occludens 1
